# *N*,*N*′-(Ethane-1,2-di­yl)dibenzene­sulfonamide

**DOI:** 10.1107/S1600536811030157

**Published:** 2011-08-02

**Authors:** Mohammad T. M. Al-Dajani, Jamal Talaat, Nornisah Mohamed, Madhukar Hemamalini, Hoong-Kun Fun

**Affiliations:** a School of Pharmaceutical Sciences, Universiti Sains Malaysia, 11800 USM, Penang, Malaysia; b Chemistry School, Virginia Commonwealth University, USA; c X-ray Crystallography Unit, School of Physics, Universiti Sains Malaysia, 11800 USM, Penang, Malaysia

## Abstract

In the title compound, C_14_H_16_N_2_O_4_S_2_, the dihedral angle between the terminal phenyl rings is 77.07 (13)°. The geometries around the S atoms are distorted tetra­hedral, with O—S—O angles of 120.66 (12) and 119.44 (11)°. In the crystal, mol­ecules are stacked in columns along the *a* axis *via* inter­molecular N—H⋯O and C—H⋯O hydrogen bonds.

## Related literature

For biological activities and applications of sulfonamide derivatives, see: Misra *et al.* (1982 ▶); Maren (1976 ▶); Li *et al.* (1995 ▶); Yoshino *et al.* (1992 ▶). For related structures, see: Basak *et al.* (1982 ▶); Cotton & Stokley (1970 ▶).
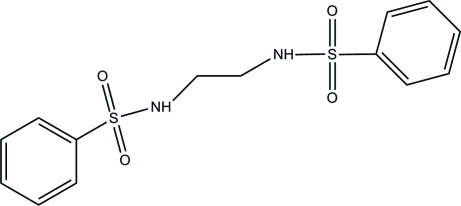


## Experimental

### 

#### Crystal data

C_14_H_16_N_2_O_4_S_2_
*M* *_r_* = 340.41
Monoclinic, 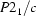

*a* = 5.2115 (4) Å
*b* = 16.6905 (13) Å
*c* = 17.8750 (14) Å
β = 93.187 (2)°
*V* = 1552.4 (2) Å^3^
*Z* = 4
Mo *K*α radiation
μ = 0.36 mm^−1^
*T* = 296 K
0.46 × 0.08 × 0.07 mm

#### Data collection

Bruker APEXII DUO CCD area-detector diffractometer
Absorption correction: multi-scan (*SADABS*; Bruker, 2009 ▶) *T*_min_ = 0.852, *T*_max_ = 0.975
14604 measured reflections
3545 independent reflections
2628 reflections with *I* > 2σ(*I*)
*R*_int_ = 0.047

#### Refinement

*R*[*F* ^2^ > 2σ(*F* ^2^)] = 0.042
*wR*(*F* ^2^) = 0.119
*S* = 1.04
3545 reflections
207 parameters
H atoms treated by a mixture of independent and constrained refinement
Δρ_max_ = 0.28 e Å^−3^
Δρ_min_ = −0.33 e Å^−3^

### 

Data collection: *APEX2* (Bruker, 2009 ▶); cell refinement: *SAINT* (Bruker, 2009 ▶); data reduction: *SAINT*; program(s) used to solve structure: *SHELXTL* (Sheldrick, 2008 ▶); program(s) used to refine structure: *SHELXTL*; molecular graphics: *SHELXTL*; software used to prepare material for publication: *SHELXTL* and *PLATON* (Spek, 2009 ▶).

## Supplementary Material

Crystal structure: contains datablock(s) global, I. DOI: 10.1107/S1600536811030157/is2755sup1.cif

Structure factors: contains datablock(s) I. DOI: 10.1107/S1600536811030157/is2755Isup2.hkl

Supplementary material file. DOI: 10.1107/S1600536811030157/is2755Isup3.cml

Additional supplementary materials:  crystallographic information; 3D view; checkCIF report

## Figures and Tables

**Table 1:** Hydrogen-bond geometry (Å, °)

*D*—H⋯*A*	*D*—H	H⋯*A*	*D*⋯*A*	*D*—H⋯*A*
N1—H1N1⋯O2^i^	0.73 (3)	2.40 (3)	3.053 (3)	149 (3)
N2—H1N2⋯O3^i^	0.83 (3)	2.15 (3)	2.924 (3)	157 (2)
C10—H10*A*⋯O1^ii^	0.93	2.57	3.294 (3)	135

Symmetry codes: (i) 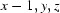; (ii) 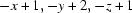.

**Fig. 1.**
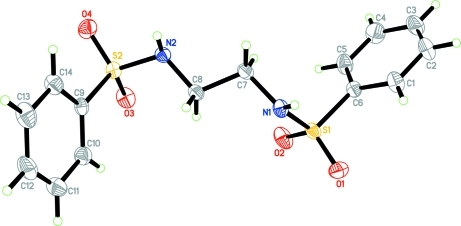
The molecular structure of the title compound, showing 50% probability displacement ellipsoids.

**Fig. 2.**
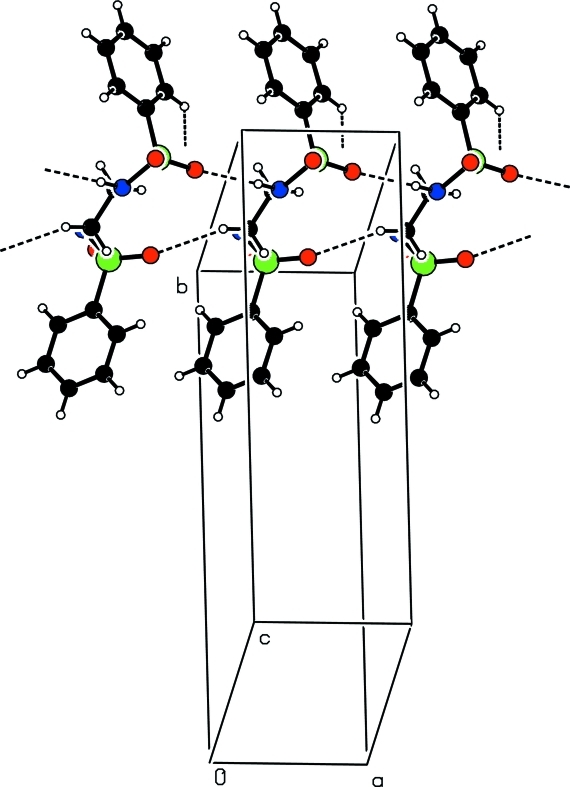
The crystal packing of the title compound with dashed lines representing hydrogen bonds.

## supplementary crystallographic information

### Comment

Sulfonamide is found in a number of synthetic as well as natural compounds. These molecules exhibit antibacterial (Misra *et al.*, 1982), insulin-releasing (Maren, 1976), anti-inflammatory (Li *et al.*, 1995) and antitumor (Yoshino *et al.*, 1992) activities.  An X-ray study of the title compound was undertaken in order to determine its crystal and molecular structure owing to the biological importance of its analogues. The molecular structure is shown in Fig. 1.

The molecule is bent at the N atoms with C9-S2-N2-C8 and C7-N1-S1-C6 torsion angles of 58.48 (18) and 72.6 (2)°, respectively. The geometries around the sulfonamide S atoms are in a slightly distorted tetrahedral configuration, similar to that observed in other reported structures (Basak *et al.*, 1982). The maximum and minimum values of the angles around S are 121.62 (17) and 105.92 (11)°, respectively. This deviation can be attributed to the non-bonded interactions involving the  S–O bonds, resulting in a structure with less steric interference (Cotton & Stokley, 1970) and the varied steric bulk of the substituents. The dihedral angle between the terminal phenyl C1–C6 and C9–C14 rings is 77.07 (13)°.

In the crystal structure, the molecules are connected *via* intermolecular N1—H1N1···O2, N2—H1N2···O3 and C10—H10A···O1 hydrogen bonds (Table 1) forming  one-dimensional supramolecular chains along the *a* axis (Fig. 2).

### Experimental

In a round bottom flask, 25ml from toluene was mixed with benzenesulfonyl chloride (0.02 mol, 3.5 g) with stirring.  Drops of ethylenediamine (0.01 mol, 0.5 g ) was added and the mixture was refluxed for 30 min. The yellow gum formed was dissolved in hot water and sodium bicarbonate was added. The yellow precipitate formed was dissolved in methanol at 60 °C, yielding colourless crystals.

### Refinement

Atoms H1N1 and H1N2 were located from a difference Fourier map and refined freely [N—H = 0.73 (3)–0.82 (3) Å]. The remaining H atoms were positioned geometrically (C—H = 0.93–0.97 Å) and were refined using a riding model, with *U*_iso_(H) = 1.2*U*_eq_(C).

### Crystal data

**Table:**

C_14_H_16_N_2_O_4_S_2_	*F*(000) = 712
*M**_r_* = 340.41	*D*_x_ = 1.456 Mg m^−^^3^
Monoclinic, *P*2_1_/*c*	Mo *K*α radiation, λ = 0.71073 Å
Hall symbol: -P 2ybc	Cell parameters from 3372 reflections
*a* = 5.2115 (4) Å	θ = 2.4–32.2°
*b* = 16.6905 (13) Å	µ = 0.36 mm^−^^1^
*c* = 17.8750 (14) Å	*T* = 296 K
β = 93.187 (2)°	Needle, colourless
*V* = 1552.4 (2) Å^3^	0.46 × 0.08 × 0.07 mm
*Z* = 4	

### Data collection

**Table:**

Bruker APEXII DUO CCD area-detector diffractometer	3545 independent reflections
Radiation source: fine-focus sealed tube	2628 reflections with *I* > 2σ(*I*)
graphite	*R*_int_ = 0.047
φ and ω scans	θ_max_ = 27.5°, θ_min_ = 1.7°
Absorption correction: multi-scan (*SADABS*; Bruker, 2009)	*h* = −6→6
*T*_min_ = 0.852, *T*_max_ = 0.975	*k* = −21→21
14604 measured reflections	*l* = −23→23

### Refinement

**Table:**

Refinement on *F*^2^	Primary atom site location: structure-invariant direct methods
Least-squares matrix: full	Secondary atom site location: difference Fourier map
*R*[*F*^2^ > 2σ(*F*^2^)] = 0.042	Hydrogen site location: inferred from neighbouring sites
*wR*(*F*^2^) = 0.119	H atoms treated by a mixture of independent and constrained refinement
*S* = 1.04	*w* = 1/[σ^2^(*F*_o_^2^) + (0.062*P*)^2^ + 0.174*P*] where *P* = (*F*_o_^2^ + 2*F*_c_^2^)/3
3545 reflections	(Δ/σ)_max_ = 0.001
207 parameters	Δρ_max_ = 0.28 e Å^−^^3^
0 restraints	Δρ_min_ = −0.33 e Å^−^^3^

### Special details

**Table:**

Geometry. All s.u.'s (except the s.u. in the dihedral angle between two l.s. planes) are estimated using the full covariance matrix. The cell s.u.'s are taken into account individually in the estimation of s.u.'s in distances, angles and torsion angles; correlations between s.u.'s in cell parameters are only used when they are defined by crystal symmetry. An approximate (isotropic) treatment of cell s.u.'s is used for estimating s.u.'s involving l.s. planes.
Refinement. Refinement of F^2^ against ALL reflections. The weighted R-factor wR and goodness of fit S are based on F^2^, conventional R-factors R are based on F, with F set to zero for negative F^2^. The threshold expression of F^2^ > 2σ(F^2^) is used only for calculating R-factors(gt) etc. and is not relevant to the choice of reflections for refinement. R-factors based on F^2^ are statistically about twice as large as those based on F, and R- factors based on ALL data will be even larger.

### Fractional atomic coordinates and isotropic or equivalent isotropic displacement parameters (Å^2^)

**Table:**

	*x*	*y*	*z*	*U*_iso_*/*U*_eq_	
S1	0.29124 (10)	0.88198 (3)	0.48674 (3)	0.03665 (17)	
S2	0.52329 (10)	0.99919 (3)	0.80016 (3)	0.03343 (16)	
O1	0.2344 (4)	0.92155 (10)	0.41688 (9)	0.0546 (5)	
O2	0.5474 (3)	0.88119 (11)	0.51993 (11)	0.0563 (5)	
O3	0.7660 (3)	0.98466 (10)	0.76908 (10)	0.0484 (4)	
O4	0.4855 (4)	0.97572 (10)	0.87531 (9)	0.0514 (5)	
N1	0.1146 (4)	0.92431 (11)	0.54596 (11)	0.0349 (4)	
N2	0.3085 (4)	0.95274 (10)	0.74743 (10)	0.0330 (4)	
C1	−0.0240 (5)	0.76368 (15)	0.43026 (14)	0.0502 (6)	
H1A	−0.1124	0.8043	0.4042	0.060*	
C2	−0.1056 (6)	0.68534 (16)	0.42342 (16)	0.0595 (7)	
H2A	−0.2505	0.6731	0.3929	0.071*	
C3	0.0258 (6)	0.62555 (15)	0.46139 (17)	0.0606 (8)	
H3A	−0.0296	0.5728	0.4560	0.073*	
C4	0.2370 (7)	0.64248 (16)	0.50704 (16)	0.0643 (8)	
H4A	0.3244	0.6015	0.5328	0.077*	
C5	0.3216 (5)	0.72138 (15)	0.51494 (14)	0.0518 (6)	
H5A	0.4654	0.7334	0.5461	0.062*	
C6	0.1905 (4)	0.78130 (13)	0.47623 (11)	0.0353 (5)	
C7	0.1430 (4)	0.90629 (12)	0.62583 (12)	0.0368 (5)	
H7A	0.2338	0.8559	0.6332	0.044*	
H7B	−0.0255	0.9006	0.6457	0.044*	
C8	0.2890 (5)	0.97194 (13)	0.66725 (11)	0.0370 (5)	
H8A	0.4596	0.9768	0.6486	0.044*	
H8B	0.2006	1.0226	0.6593	0.044*	
C9	0.4584 (4)	1.10252 (12)	0.79155 (11)	0.0325 (5)	
C10	0.5938 (5)	1.14968 (14)	0.74477 (13)	0.0451 (6)	
H10A	0.7253	1.1279	0.7181	0.054*	
C11	0.5318 (6)	1.23066 (15)	0.73773 (15)	0.0557 (7)	
H11A	0.6214	1.2632	0.7059	0.067*	
C12	0.3395 (6)	1.26242 (15)	0.77743 (16)	0.0566 (7)	
H12A	0.3001	1.3166	0.7730	0.068*	
C13	0.2043 (6)	1.21465 (16)	0.82375 (17)	0.0592 (7)	
H13A	0.0721	1.2366	0.8500	0.071*	
C14	0.2625 (5)	1.13442 (14)	0.83181 (14)	0.0456 (6)	
H14A	0.1720	1.1022	0.8637	0.055*	
H1N1	−0.017 (5)	0.9291 (15)	0.5307 (14)	0.034 (7)*	
H1N2	0.168 (5)	0.9581 (14)	0.7659 (13)	0.036 (6)*	

### Atomic displacement parameters (Å^2^)

**Table:**

	*U*^11^	*U*^22^	*U*^33^	*U*^12^	*U*^13^	*U*^23^
S1	0.0300 (3)	0.0369 (3)	0.0434 (3)	−0.0052 (2)	0.0060 (2)	−0.0049 (2)
S2	0.0273 (3)	0.0334 (3)	0.0391 (3)	0.0054 (2)	−0.0026 (2)	−0.0020 (2)
O1	0.0715 (13)	0.0515 (10)	0.0419 (9)	−0.0085 (9)	0.0125 (9)	0.0041 (7)
O2	0.0266 (9)	0.0588 (11)	0.0835 (12)	−0.0066 (8)	0.0036 (9)	−0.0167 (9)
O3	0.0240 (8)	0.0516 (10)	0.0694 (11)	0.0088 (7)	−0.0001 (8)	−0.0077 (8)
O4	0.0628 (12)	0.0508 (9)	0.0397 (9)	0.0058 (9)	−0.0064 (8)	0.0064 (7)
N1	0.0268 (10)	0.0378 (10)	0.0397 (10)	0.0006 (8)	−0.0024 (9)	−0.0045 (8)
N2	0.0276 (10)	0.0339 (9)	0.0378 (9)	−0.0010 (8)	0.0057 (8)	−0.0022 (7)
C1	0.0468 (15)	0.0431 (13)	0.0595 (15)	−0.0009 (12)	−0.0085 (13)	−0.0065 (11)
C2	0.0548 (17)	0.0508 (15)	0.0714 (18)	−0.0129 (13)	−0.0094 (15)	−0.0140 (13)
C3	0.075 (2)	0.0389 (13)	0.0684 (17)	−0.0122 (14)	0.0061 (16)	−0.0111 (12)
C4	0.086 (2)	0.0411 (13)	0.0645 (17)	0.0081 (15)	−0.0052 (17)	0.0018 (12)
C5	0.0519 (16)	0.0467 (13)	0.0550 (14)	0.0048 (12)	−0.0123 (13)	−0.0053 (11)
C6	0.0315 (12)	0.0365 (11)	0.0383 (11)	0.0003 (9)	0.0052 (10)	−0.0064 (9)
C7	0.0366 (12)	0.0330 (10)	0.0408 (11)	−0.0065 (9)	0.0033 (10)	−0.0018 (9)
C8	0.0388 (12)	0.0333 (10)	0.0385 (11)	−0.0073 (10)	−0.0012 (10)	0.0005 (9)
C9	0.0285 (11)	0.0341 (10)	0.0340 (10)	0.0012 (9)	−0.0059 (9)	−0.0059 (8)
C10	0.0467 (14)	0.0427 (12)	0.0462 (13)	0.0003 (11)	0.0056 (11)	−0.0038 (10)
C11	0.0676 (19)	0.0419 (13)	0.0573 (15)	−0.0067 (13)	0.0012 (14)	0.0049 (11)
C12	0.0651 (19)	0.0312 (12)	0.0719 (17)	0.0059 (12)	−0.0101 (15)	−0.0042 (11)
C13	0.0524 (16)	0.0446 (14)	0.0805 (19)	0.0124 (13)	0.0043 (15)	−0.0164 (13)
C14	0.0392 (13)	0.0401 (12)	0.0581 (14)	0.0039 (10)	0.0093 (12)	−0.0083 (10)

### Geometric parameters (Å, °)

**Table:**

S1—O1	1.4291 (17)	C4—H4A	0.9300
S1—O2	1.4307 (19)	C5—C6	1.376 (3)
S1—N1	1.605 (2)	C5—H5A	0.9300
S1—C6	1.767 (2)	C7—C8	1.505 (3)
S2—O4	1.4233 (17)	C7—H7A	0.9700
S2—O3	1.4300 (17)	C7—H7B	0.9700
S2—N2	1.6202 (19)	C8—H8A	0.9700
S2—C9	1.763 (2)	C8—H8B	0.9700
N1—C7	1.459 (3)	C9—C10	1.372 (3)
N1—H1N1	0.73 (3)	C9—C14	1.387 (3)
N2—C8	1.467 (3)	C10—C11	1.394 (3)
N2—H1N2	0.82 (3)	C10—H10A	0.9300
C1—C2	1.378 (3)	C11—C12	1.367 (4)
C1—C6	1.382 (3)	C11—H11A	0.9300
C1—H1A	0.9300	C12—C13	1.372 (4)
C2—C3	1.369 (4)	C12—H12A	0.9300
C2—H2A	0.9300	C13—C14	1.379 (3)
C3—C4	1.363 (4)	C13—H13A	0.9300
C3—H3A	0.9300	C14—H14A	0.9300
C4—C5	1.393 (4)		
			
O1—S1—O2	120.66 (12)	C5—C6—C1	120.5 (2)
O1—S1—N1	105.92 (11)	C5—C6—S1	120.09 (19)
O2—S1—N1	106.67 (11)	C1—C6—S1	119.43 (18)
O1—S1—C6	107.49 (10)	N1—C7—C8	110.63 (17)
O2—S1—C6	107.46 (11)	N1—C7—H7A	109.5
N1—S1—C6	108.11 (10)	C8—C7—H7A	109.5
O4—S2—O3	119.44 (11)	N1—C7—H7B	109.5
O4—S2—N2	106.86 (11)	C8—C7—H7B	109.5
O3—S2—N2	106.87 (10)	H7A—C7—H7B	108.1
O4—S2—C9	108.40 (10)	N2—C8—C7	109.10 (17)
O3—S2—C9	107.56 (10)	N2—C8—H8A	109.9
N2—S2—C9	107.12 (10)	C7—C8—H8A	109.9
C7—N1—S1	121.62 (17)	N2—C8—H8B	109.9
C7—N1—H1N1	115.6 (19)	C7—C8—H8B	109.9
S1—N1—H1N1	111 (2)	H8A—C8—H8B	108.3
C8—N2—S2	118.16 (14)	C10—C9—C14	120.9 (2)
C8—N2—H1N2	110.7 (16)	C10—C9—S2	120.78 (17)
S2—N2—H1N2	108.3 (16)	C14—C9—S2	118.26 (17)
C2—C1—C6	119.4 (2)	C9—C10—C11	119.1 (2)
C2—C1—H1A	120.3	C9—C10—H10A	120.4
C6—C1—H1A	120.3	C11—C10—H10A	120.4
C3—C2—C1	120.3 (3)	C12—C11—C10	120.2 (2)
C3—C2—H2A	119.9	C12—C11—H11A	119.9
C1—C2—H2A	119.9	C10—C11—H11A	119.9
C4—C3—C2	120.7 (2)	C11—C12—C13	120.3 (2)
C4—C3—H3A	119.6	C11—C12—H12A	119.9
C2—C3—H3A	119.6	C13—C12—H12A	119.9
C3—C4—C5	119.8 (3)	C12—C13—C14	120.7 (2)
C3—C4—H4A	120.1	C12—C13—H13A	119.7
C5—C4—H4A	120.1	C14—C13—H13A	119.7
C6—C5—C4	119.4 (3)	C13—C14—C9	118.8 (2)
C6—C5—H5A	120.3	C13—C14—H14A	120.6
C4—C5—H5A	120.3	C9—C14—H14A	120.6
			
O1—S1—N1—C7	−172.40 (17)	N1—S1—C6—C1	80.2 (2)
O2—S1—N1—C7	−42.7 (2)	S1—N1—C7—C8	102.1 (2)
C6—S1—N1—C7	72.6 (2)	S2—N2—C8—C7	162.43 (15)
O4—S2—N2—C8	174.49 (16)	N1—C7—C8—N2	178.68 (19)
O3—S2—N2—C8	−56.57 (19)	O4—S2—C9—C10	145.79 (19)
C9—S2—N2—C8	58.48 (18)	O3—S2—C9—C10	15.4 (2)
C6—C1—C2—C3	−0.5 (4)	N2—S2—C9—C10	−99.2 (2)
C1—C2—C3—C4	0.7 (5)	O4—S2—C9—C14	−35.8 (2)
C2—C3—C4—C5	−0.4 (5)	O3—S2—C9—C14	−166.27 (18)
C3—C4—C5—C6	−0.2 (4)	N2—S2—C9—C14	79.1 (2)
C4—C5—C6—C1	0.4 (4)	C14—C9—C10—C11	−0.2 (4)
C4—C5—C6—S1	178.9 (2)	S2—C9—C10—C11	178.08 (19)
C2—C1—C6—C5	−0.1 (4)	C9—C10—C11—C12	0.4 (4)
C2—C1—C6—S1	−178.6 (2)	C10—C11—C12—C13	−0.8 (4)
O1—S1—C6—C5	147.7 (2)	C11—C12—C13—C14	0.9 (4)
O2—S1—C6—C5	16.4 (2)	C12—C13—C14—C9	−0.7 (4)
N1—S1—C6—C5	−98.4 (2)	C10—C9—C14—C13	0.4 (4)
O1—S1—C6—C1	−33.8 (2)	S2—C9—C14—C13	−178.0 (2)
O2—S1—C6—C1	−165.05 (19)		

### Hydrogen-bond geometry (Å, °)

**Table:**

*D*—H···*A*	*D*—H	H···*A*	*D*···*A*	*D*—H···*A*
N1—H1N1···O2^i^	0.73 (3)	2.40 (3)	3.053 (3)	149 (3)
N2—H1N2···O3^i^	0.83 (3)	2.15 (3)	2.924 (3)	157 (2)
C10—H10A···O1^ii^	0.93	2.57	3.294 (3)	135.

Symmetry codes: (i) *x*−1, *y*, *z*; (ii) −*x*+1, −*y*+2, −*z*+1.
